# Beyond Ce–U Charge Transfer: Strain and Polarons in Epitaxial Ce_1–_ *_x_* U*_x_* O_2_(111)

**DOI:** 10.1021/acs.jpcc.6c05108

**Published:** 2026-09-17

**Authors:** Carlos Morales, Rudi Tschammer, Aman Baunthiyal, Thomas Gouder, Jan Ingo Flege, Hicham Idriss

**Affiliations:** † Applied Physics and Semiconductor Spectroscopy, Brandenburg University of Technology Cottbus−Senftenberg, Konrad-Zuse-Strasse 1, D-03046 Cottbus, Germany; ‡ Institute of Solid State Physics, 9168 University of Bremen, D-28359 Bremen, Germany; § European Commission, Joint Research Centre (JRC), Postfach 2340, D-76125 Karlsruhe, Germany; ∥ Department of Chemistry, 150232 University College London, UCL, London WC1E 6BT, U.K.

## Abstract

The redox chemistry of mixed ceria-based oxides is commonly interpreted in terms of direct charge transfer between neighboring cations. In the Ce_1–_ *_x_* U*_x_* O_2_ system, this corresponds to the oxidation of U^4+^ into U^5+^ accompanied by the reduction of Ce^4+^ into Ce^3+^. In this work, epitaxial Ce_1–_ *_x_* U*_x_* O_2_(111) thin films with different uranium contents (with x ranging from 0.05 to 1) were grown under ultra-high vacuum conditions and investigated using angle-resolved X-ray photoelectron spectroscopy. The measurements reveal a substantial excess of Ce^3+^ relative to U^5+^, particularly at low uranium fractions and in the topmost surface region, indicating that the reduction of Ce^4+^ cations cannot be explained solely by direct U^4+^ to Ce^4+^ charge transfer. Quantitative analysis further shows deviations in oxygen stoichiometry when lattice oxygen (O_*l*_) is assumed to remain entirely in the O^2–^ state. The effect is stronger in epitaxial films and largely absent in structurally disordered counterparts. These observations support a picture in which oxygen ions participate in the redox process through lattice distortion and polaron-mediated charge redistribution. The results suggest that (i) oxygen-mediated electronic coupling, (ii) strain, and (iii) local structural order collectively govern the reduction behavior in Ce_1–_ *_x_* U*_x_* O_2_(111), with important implications for ceria-based redox materials used in catalysis and thermochemical fuel production.

## Graphical abstract

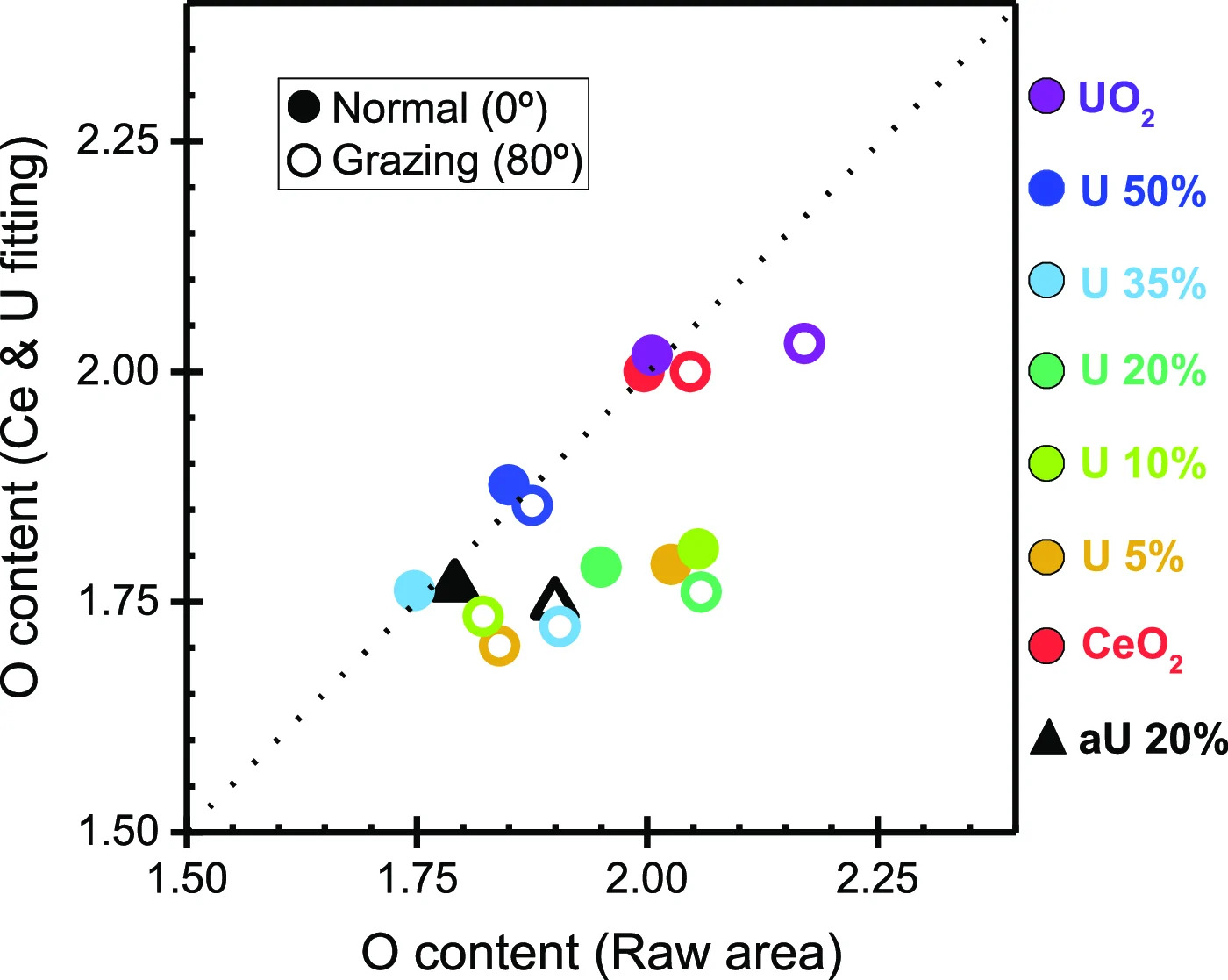

## 1 Introduction

Cerium dioxide (CeO_2_) is one of the most studied oxides in catalysis and related materials. This is largely due to its stability and redox properties, both of which make it an important support for many metals in numerous catalytic processes, spanning from the water–gas shift reaction (WGSR),^1^ hydrocarbons oxidation,^2^ and carbon–carbon bond formation,^3–5^ among others. It is also a benchmark oxide in thermochemical water splitting^6^ (TCWS) and CO_2_-to-CO thermal reduction.^7^ For these reactions, the presence of reduced Ce cations (Ce^3+^) as a consequence of oxygen vacancy formation is essential (CeO_2‑x_), requiring the removal of up to 15% of oxygen atoms, i.e., CeO_1.7_.

Ceria reduction is typically achieved in catalysis using reducing agents, such as H_2_ in WGSR (CO_2_ + H_2_ → CO + H_2_O), or by hydrocarbons in automobile catalytic conversion^8^ or ethylbenzene dehydrogenation.^9^ These redox reactions generally work well because an active metal phase at the oxide surface enables rapid reaction rates and high turnover frequency. However, such an extent of reduction is not suitable for the TCWS (H_2_O →ΔH_2_ + 1/2 O_2_) or thermal CO_2_ reduction (CO_2_ →Δ CO + 1/2 O_2_) processes, which rely on two steps: first thermal reduction of ceria upon heating to high temperature in an inert environment (typically >1500 ^°^C), and subsequent reoxidation through exposure to water vapor (or CO_2_) and concomitant O–H (or C=O) bond dissociation. This process structure substantially limits the reaction’s efficiency, with complete cycle times extending from minutes to an hour. Numerous experimental,^10–12^ computational,^13–15^ and techno-economic^16^ works have focused on these drawbacks to increase the extent of reduction. In that regard, numerous strategies have been pursued, including mixing with other metal cations to reduce the oxygen vacancy formation energy^17–20^ (*E*_VO_) and thereby increasing the reaction yield. Albeit introducing additional complications, as the mixed (doped) oxide must withstand numerous cycles without phase segregation, the decrease in *E*_VO_ lowers the thermal reduction temperature and increases the number of reduced sites.

Among the many strategies to enhance the CeO_2_ reduction by mixing it with other metal cations, the following three are the most common. First, substituting a fraction of Ce^4+^ cations (typically less than half) by non-reducible metal cations of smaller ionic radii compared to that of Ce^4+^ (0.97 Å) (e.g., Zr^4+^ with a size of 0.84 Å for an eight-fold coordination).^21^ The induced lattice strain is relaxed through lattice expansion upon the formation of Ce^3+^ (1.14 Å). Second, the substitution of a fraction of Ce^4+^ cations by metal cations with lower oxidation states and with larger^22^ or smaller^23^ sizes (e.g., Sm^3+^ (1.08 Å) or Fe^3+^ (0.65 Å)). This aliovalent substitution primarily introduces extrinsic oxygen vacancies, i.e., those solely induced by charge compensation to maintain electrical neutrality, and does not, strictly speaking, constitute a reduction process. However, the resulting defect structure lowers the energy required for subsequent oxygen removal, thereby facilitating the reduction of Ce^4+^ to Ce^3+^ under reducing conditions.^22^ Finally, substituting a fraction of Ce^4+^ with oxidizable metal cations (e.g., V^4+^ or U^4+^), which induces a charge transfer from the dopant to Ce^4+^ (making Ce^3+^ and M^5+^).

Common to the three methods mentioned above is local structural disorder, as reflected in the distribution of dopant cations within the lattice and induced local strain. Ceria doping with uranium illustrates this complex interplay. U^4+^ cations are ideal dopants for ceria:^24^ (i) a U^4+^ cation has a size almost identical to that of Ce^4+^, thus minimizing lattice distortion, (ii) CeO_2_ and UO_2_ adopt the fluorite structure, which guarantees good solubility, and (iii) they readily oxidize to U^5+^ and U^6+^ upon oxygen incorporation (UO_2+δ_). Indeed, several works have shown a relationship between the degree of reduction of Ce^4+^ in Ce_1–*x*_U_*x*_O_*y*_ and the amount of H_2_ produced by thermal water splitting.^25,26^ However, it was also found computationally, in line with others, that the distribution of U^4+^ and Ce^4+^ cations critically affects the charge transfer, which in turn alters the lattice by local asymmetric distortion of the U–O and C–O bonds.^26,27^ Furthermore, recent experiments involving epitaxial Ce_1–*x*_U_*x*_O_*y*_ (111)-oriented films showed a large mismatch between the amount of U^5+^ cations formed and the amount of Ce^3+^, with the latter being manyfold higher than the former, particularly at low U concentrations.^26^ In addition to the entropic factor previously noted regarding elemental distribution, i.e., local arrangement of Ce and U cations, and charge disorder, i.e., presence and local distribution of cations with different oxidation states,^26,27^ another reason must be the change in the size of U cations upon oxidation to +5 (0.84 Å) and the role of local lattice distortion. In short, it appears that structural distortion is the common kinetic factor behind the thermal reduction of CeO_2_ in the presence of U dopant (which probably can be generalized to other mixed oxides).

These observations have led us to re-examine the Ce_1–*x*_U_*x*_O_*y*_ system and focus on the reasons for deviations from the expected Ce–U charge-transfer. Compared to more realistic powder samples, the study of model systems using surface science approaches enables a more direct characterization of the interplay among structure, composition, and cation oxidation state, as well as a more straightforward comparison with computational studies. Here, we have studied Ce_1–*x*_U_*x*_O_2±δ_ (111)-oriented films with different U concentrations by X-ray photoelectron spectroscopy (XPS). In CeO_2_, the (111) facet is the most stable surface orientation,^28^ where topmost oxygen anions are three-fold coordinated to underlying Ce^4+^ cations and the latter are seven-fold coordinated to O^2–^. The removal of an oxygen atom from the surface leaves behind two electrons that are captured by two Ce^4+^ cations, resulting in the formation of two Ce^3+^. The location of the removed oxygen at the topmost atomic layer or beneath has been computationally studied in great detail.^29–32^ It has been shown to depend on multiple parameters, including surface termination, the number of oxygen atoms to be removed (i.e., the oxygen-defect density), and lattice strain in the presence or absence of a dopant (in the case of a mixed oxide). Experimentally, a variety of techniques, including core-level spectroscopies,^33–37^ electron paramagnetic resonance (EPR),^38^ and Raman spectroscopy,^39^ are used to indirectly probe oxygen vacancies by monitoring signals associated with Ce^3+^ cations. In the surface and near-surface regions, photoelectron spectroscopies are well suited to study the Ce 3d core-level and valence-band region (i.e., Ce 4f).

While the above wealth of information on the electronic structure and properties of CeO_2_ in the presence of dopants has considerably increased our understanding of its redox properties, the quantitative experimental results on the oxidation states of Ce and the dopants in a well-structured mixed oxide are still missing. In that regard, our detailed quantitative XPS study of the surface and near-surface of Ce_1–*x*_U_*x*_O_2_ (111) epitaxial mixed oxide films provides a clearer picture of the reasons behind the enhanced reduction of Ce^4+^ to Ce^3+^.

## 2 Experimental Section

### 2.1 Growth of Epitaxy Ce_1–*x*_U_*x*_O_2_

Ultrasmooth Ru(0001) thin films were grown by magnetron sputtering on sapphire (0001) substrates with a misorientation of less than 0.1° (Crystec) under an Ar atmosphere of ∼5 × 10^–3^ mbar and at a sample temperature of 600 °C. The deposition process is thoroughly described elsewhere^26^ and follows a similar approach to that reported by several groups.^40,41^ The quality of the Ru(0001) epitaxial thin films, in terms of homogeneity and degree of crystallinity, was checked by low-energy electron microscopy (LEEM) using a commercial Elmitec LEEM III system. Amorphous Ru thin films were deposited on SiO_2_/Si substrates under the same growth conditions for comparison purposes.

The *in-situ* growth and characterization of epitaxial Ce_1–x_U_*x*_O_2_(111) thin films was carried out at the EU Joint Research Center, laboratory of Properties of Actinide Materials under Extreme Conditions (PAMEC, ActUsLab). The Ru(0001)/Al_2_O_3_(0001) and Ru/SiO_2_ templates were transported under rough vacuum (∼10^–1^ mbar) to the JRC and transferred to the PAMEC ultra-high vacuum (UHV) cluster through air. Prior to the deposition of epitaxial Ce_1–x_U*_x_* O_2_ (111) films (i.e., *x* = 0, 0.05, 0.10, 0.20, 0.35, 0.50, and 1), the Ru thin films were cleaned by several annealing cycles to 600 °C in an oxygen background pressure (5 × 10^–7^ mbar), which was pumped down to <10^–9^ mbar during cooling when the temperature reached 400 °C. The surface cleanliness and quality were subsequently confirmed by low-energy electron diffraction (LEED) and X-ray photoelectron spectroscopy (XPS). The epitaxial Ce_1–x_U_*x*_O_2_ (111) thin films were prepared *in-situ* at a substrate temperature of 400 °C by direct current (DC) sputtering from *metallic* cerium and uranium targets in a gas mixture of Ar (6N) and O_2_ (6N), with a total pressure of ∼6 × 10^–3^ mbar and an O_2_ partial pressure of 10^–6^ mbar. The nominal Ce/U ratio was achieved by varying the target voltages and emission currents, keeping the total power constant (ca. 1 W) and establishing a deposition rate of about 7 Å/min. All samples have a total thickness of about 8 nm. For comparison purposes, an amorphous (nanocrystalline) Ce_0.8_U_0.2_O_2_ was grown on an amorphous Ru substrate (Ru/SiO_2_) under the same conditions. Immediately after growth, the as-grown samples were transferred under UHV conditions to the analysis chamber within the same surface science cluster for characterization.

### 2.2 Characterization of Epitaxy Ce_1–*x*_U*_x_* O_2_

The whole *in-situ* characterization of Ce_1–x_U*_x_* O_2±δ_ thin films was performed at room temperature in the analysis chamber of the JRC UHV-cluster, which presents a background pressure of 2 × 10^–10^ mbar. An ErLEED (SPECS) system was used to obtain LEED patterns at electron energies between 40 and 120 eV. The XPS measurements were performed using a Phoibos 150 hemispherical (SPECS) analyzer equipped with a nine-channeltron detector and Al K_α_ radiation (*E*_hν_ = 1486.6 eV) from a micro-focus source (XRC-1000) operating at 120 W and equipped with a monochromator. The spectra were measured at two different take-off angles: 0 and 80°. The overall energy resolution was about 0.55 eV using a pass energy of 20 eV. The spectrometer was initially calibrated using the Au 4f_7/2_ (83.9 eV binding energy) and Cu 2p_3/2_ (932.7 eV binding energy) peaks from clean metallic Au and Cu surfaces, respectively.^42^ Sample charging was corrected by employing an electron flood gun (FG 22/35, SPECS) and cross-checking the binding energy scale with internal references (Ru 3d_5/2_ at 280.0 eV^42^ and CeO_2_ u’’’ component at 917.0 eV^43^). The XPS data were handled using CasaXPS 2.3 and XPSpeak 4.2 software. The sample composition was estimated using the specific atomic sensitivity factors of the XPS analyzer, which were cross-checked against stoichiometric reference samples of CeO_2_ and UO_2_. Moreover, the inelastic mean free path of photoemitted electrons through the Ce_1–*x*_U*_x_* O_2±δ_ matrix has been estimated using the Tanuma, Powell, and Penn formula IMFP-TPP2M^44^ implemented in the QUASES-IMFP software. Inelastic peak shape analysis (IPSA) was carried out using the QUASES-Analysis software on the O 1s extended region (up to 100 eV in binding energy above the elastic peak).^45,46^

After Shirley background removal, the experimental Ce 3d and U 4f spectra were fitted using a target-testing factor analysis (FA) approach using MATLAB, with reference spectra for Ce 3d (CeO_2_ and Ce_2_O_3_) and U 4f (UO_2_ and U_2_O_5_).^47^ FA was preferred over other methods due to its ability to directly derive the main spectral components from the collective dataset while simultaneously correcting for the inherent background difference between the experimental data and the reference spectra. This was particularly important for the Ce 3d region, for which the reference spectra were taken in a different system. Additionally, the U 4f spectra showed experimental peaks that were noticeably narrower than those in the reference spectra published by Gouder and co-authors.^47^ Figure S1 in the Supporting Information compares the reference spectra with the main components obtained in the FA process, and Figure S2 exemplifies the goodness of the analysis. An overall uncertainty of ±2% is estimated when calculating relative contents of Ce and U cations through this methodology. The O 1s region was fitted using a standard peak model comprising four components (using symmetric pseudo-Voigt functions) after Shirley background subtraction. The lattice oxygen (metal–oxygen bond, Me-O) at ca. 530 eV was treated as a single contribution, independent of variations in the Ce/U ratio or of the different oxidation states Ce^4+^/Ce^3+^ and U^4+^/U^5+^ present in the samples. This simplification provided a good fit for the data. Three additional peaks were introduced, shifted by 1.2, 2.2, and 3.0 eV relative to the Me-O component. Following previous studies on the adsorption of water on oxidized and reduced cerium oxide (111)-oriented surfaces,^48^ the components at 1.2 and 2.2 eV are treated as contributions from hydroxyl (OH^‑^) groups in oxidized and reduced CeO_2‑x_ (111) surfaces, respectively. Finally, the component at 3.0 eV is assigned to molecularly adsorbed water (H_2_O). The energy position and component full-width at half-maximum (FWHM) values were fixed for all samples.

## 3 Results and Discussion

### 3.1 As-grown Ce_1–*x*_U*_x_* O_2_ (111) Films: near Bulk vs Surface Oxidation States of Ce and U Cations

Epitaxial Ce_1–x_U_*x*_O_2_ (111) films were grown over 700 s at 400 °C and 10^–6^ mbar O_2_ partial pressure to guarantee the deposition of a continuous film fully covering the Ru substrate^49^ and with sufficient thickness to avoid potential lattice distortion due to lattice mismatch between (111)-oriented ceria and Ru(0001). Previous studies have shown that CeO_2_ (111)-oriented nano islands exhibit a commensurate structure on Ru (0001) that prevents high strain despite the lattice mismatch.^49,50^ The absence of the Ru 3d signal in the as-grown XPS survey spectra in Figure 1a, as well as the absence of the Ru diffraction spots in the LEED pattern in Figure 1b, demonstrates full substrate coverage. The inelastic peak-shape analysis (IPSA) of the O 1s extended region presented in Figure S3 further confirms the formation of a continuous layer of about 8 nm for all samples (i.e., about 26 ceria monolayers (ML), considering the thickness of an O–Ce–O trilayer of the (111)-oriented fluorite structure equal to 3.12 Å, see Table S1). Moreover, the survey spectra show only peaks associated with Ce, U, and O, with a very small peak at 680 eV indicating traces of fluorine. The latter was found to be proportional to the time the samples were stored in the transfer chamber overnight. The estimated compositions of the films from XPS measurements at 0° and 80° take-off angles (i.e., with effective probing depths, i.e., 3·λ·cosθ, of about 4.3 and 0.8 nm, respectively) is presented in Table S2 and coincides with the intended target compositions. Finally, the hexagonal diffraction pattern of the Ce_0.8_U_0.2_O_2_ sample confirms epitaxial growth along the (111) direction, whereas the faint diffraction spots and the strong background signal relative to the clean Ru substrate indicate a high density of structural defects.

**Figure 1:**
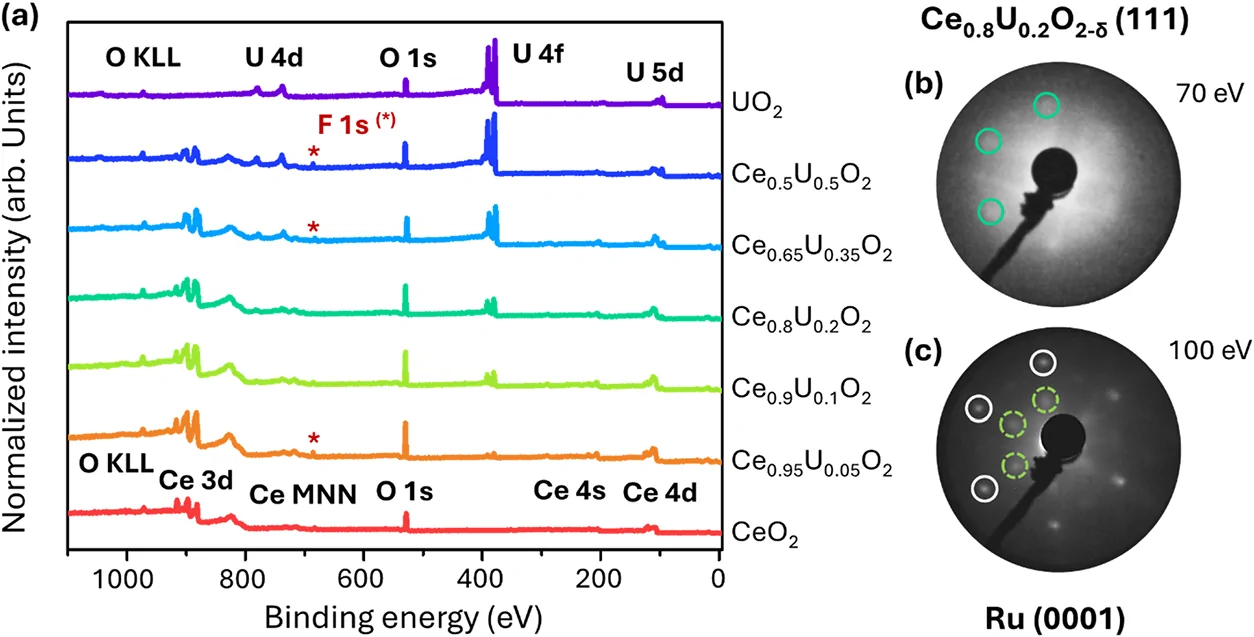
(a) XPS survey spectra of the Ce_1–*x*_U_*x*_O_2_ samples. The main regions are indicated. LEED patterns of (b) Ce_0.8_U_0.2_O_2_ (111) film and (c) clean Ru(0001) substrate at the indicated primary energy, E_p_ (eV). The hexagonal pattern indicated by solid white circles corresponds to the Ru spots, while the dashed green circles indicate the (2 × 1) reconstruction of adsorbed oxygen. Solid green circles indicate spots in azimuthal registry with the Ru(0001).

Figures 2a,b and 3a,c show the Ce 3d and U 4f spectra for the series of samples taken with take-off angles of 0 and 80^o^, respectively. For the reference CeO_2_ sample, the Ce 3d lines are dominated by Ce^4+^ cations (*v* = 882.6 eV, *v‴* = 898.7 eV, and *u‴* = 917.0 eV). For the mixed oxides, Ce 3d spectra indicate a significant fraction of Ce^3+^, as evidenced by a strong reduction of the *u‴* component and the appearance of new peaks related to *v*′ (885.7 eV) and *u*′ (904.4 eV) Ce^3+^ components.^43,51^ Changes in the U 4f spectra for different oxidation states are also clear. Briefly, the spectroscopic differences between the U^4+^ and U^5+^ oxidation states are tracked by both the binding energy position (Figure 3b) and corresponding satellites (Figure 3a).^47,52^ The U 4f_7/2_ component of U^4+^ is positioned at 380.0 eV, with a satellite at 6.9 eV above, while that of the U^5+^ sits at 381.0 eV, with a satellite at 8.4 eV above. In all mixed oxide samples, the U^5+^ oxidation state was observed, whereas the UO_2_ film alone contained only U^4+^ cations. This observation is common to both sets of spectra, measured at normal (0°) and glancing (80°) take-off angles.

**Figure 2:**
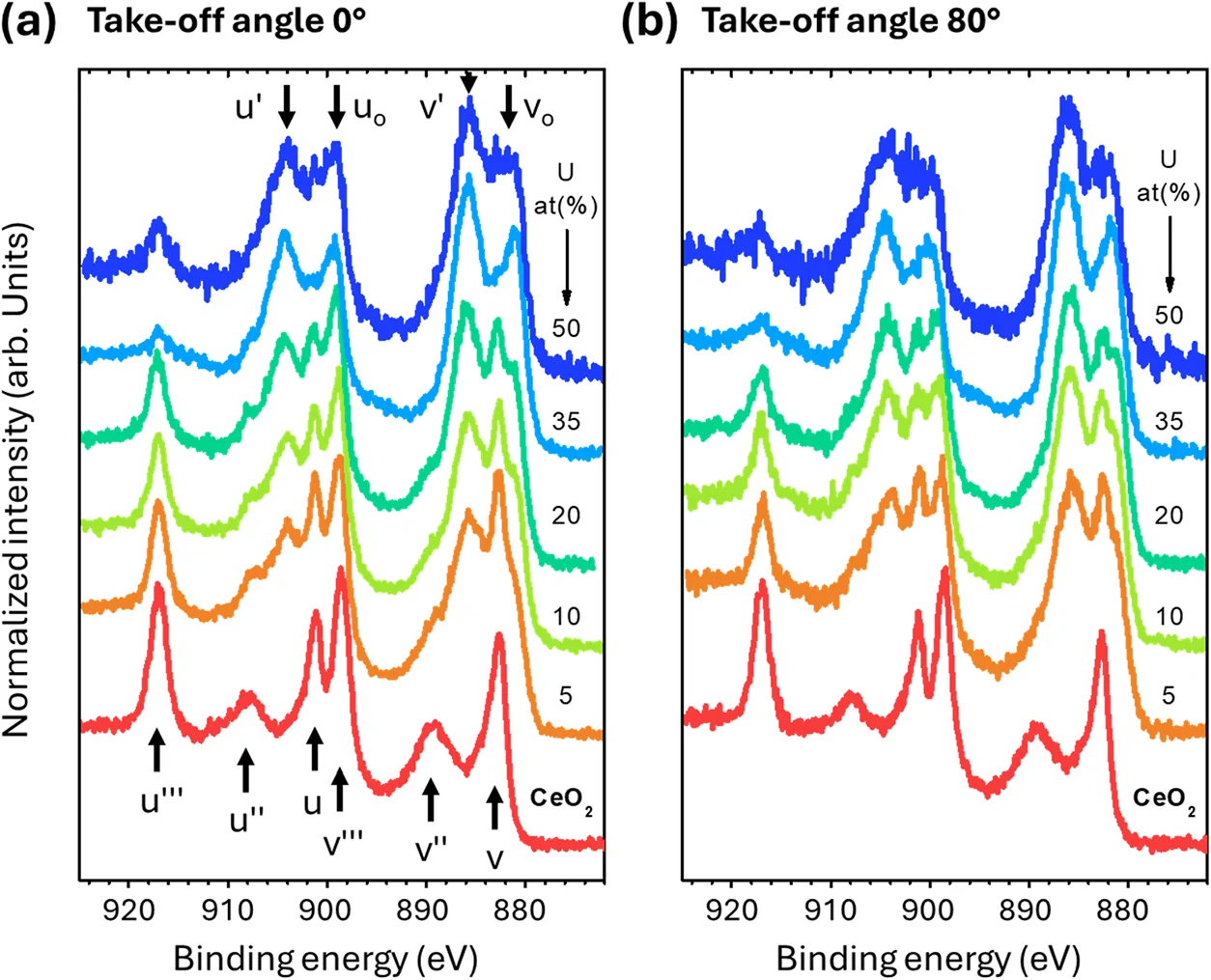
XPS Ce 3d lines of the series Ce_1–*x*_U_*x*_O_2_ at the indicated U % for measurements conducted at (a) 0^o^ and (b) 80^o^ take-off angles. At the top and bottom of panel (a), the components associated with Ce^3+^ and Ce^4+^ states are indicated, respectively. The U at(%) is taken with respect to the content of Ce.

**Figure 3:**
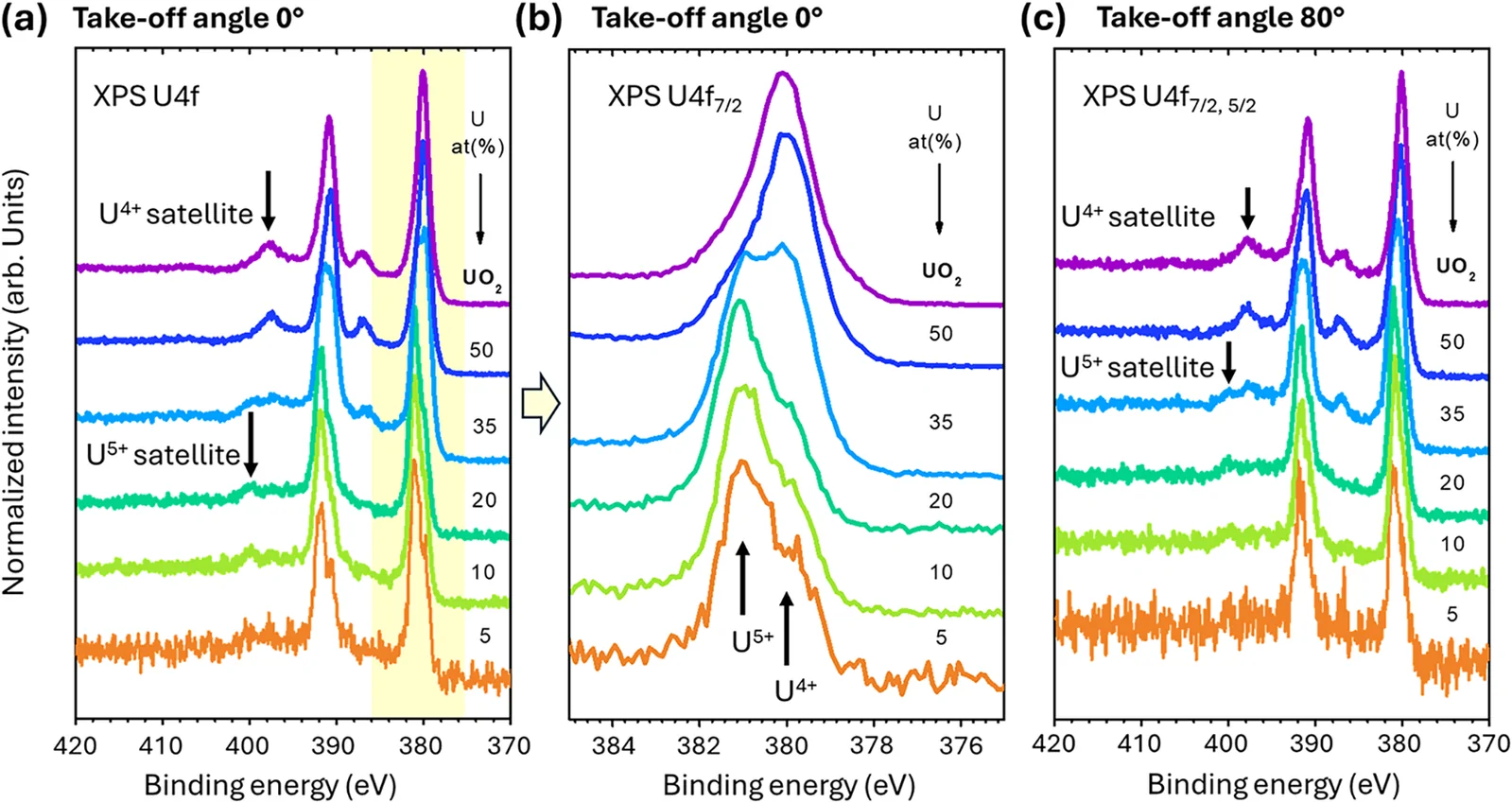
XPS U 4f lines of the series Ce_1–*x*_U_*x*_O_2_ at the indicated U % for measurements conducted at 0^o^ and 80^o^ take-off angles. (a) XPS U 4f lines of the series Ce_1–*x*_U_*x*_O_2_ at the indicated U % for measurements conducted at 0^o^. (b) Zoom of the yellow-highlighted area in (a) for a better view of the two oxidation states of U cations in the series. (c) XPS U 4f lines of the series Ce_1–*x*_U_*x*_O_2_ at the indicated U % for measurements conducted at 80^o^. The U at(%) is taken with respect to the content of Ce.

Moreover, the Ce 3d and U 4f spectra have been evaluated using a target-testing factor analysis to estimate the relative weights of the Ce^3+^/Ce^4+^ and U^4+^/U^5+^ oxidation states (Figure S2 shows the comparison between the raw data and the fit). Figure 4a,b displays the evolution of Ce^3+^ and U^5+^ states for all series of epitaxial films at near-surface and surface regions (i.e., normal and grazing take-off angles). It is seen that the amount of Ce^3+^ cations increases with the U content up to ca. 30 atom %, after which it saturates or even slightly decreases. On the other hand, the concentration of U^5+^ states gradually decreases with increasing U content. Notably, the topmost layers appear to contain greater amounts of Ce^3+^ cations (Figure 4a). For the U^5+^ states, there appears to be a reversal trend compared to Ce^3+^ cations. The U^5+^ contribution is systematically lower at the surface than in the bulk when U is present at low concentrations (Figure 4b). Therefore, the data indicate that the topmost layers have a higher contribution of Ce^3+^ and a lower contribution of U^5+^ than deeper regions.

**Figure 4:**
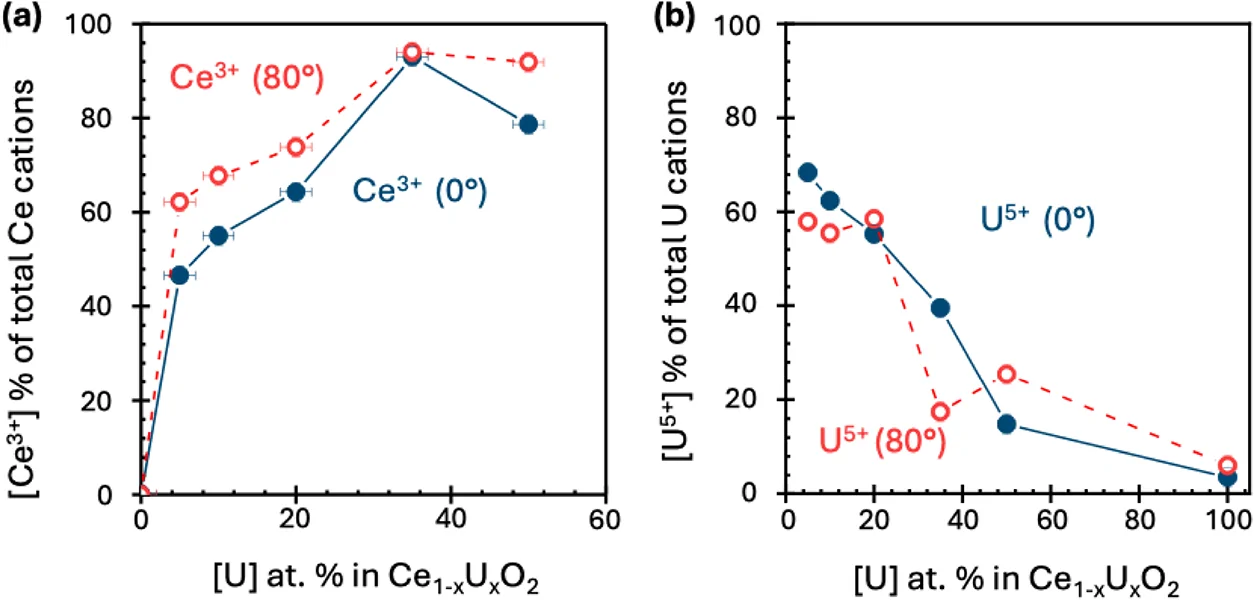
Estimated Ce^3+^ and (b) U^5+^ percentage (*y*-axes) for the Ce_1–*x*_U_*x*_O_2_ (111)-oriented series as a function of U content (*x*-axis). Blue full symbols and red open symbols refer to measurements obtained at normal (0 °) and grazing (80 °) take-off angles, respectively. The U at(%) is taken with respect to the content of Ce.

As noted above, the films are about 8 nm thick, hence much larger than the inelastic mean free path (λ) of the photoelectrons at both configurations (0 and 80°). The inelastic mean free path for Ce cations (with kinetic energy, KE, of about 600 eV when using Al K_α_ radiation) and for U cations (with KE of about 1050 eV) is estimated as 1.17 and 1.75 nm for mixed cerium–uranium oxide matrixes,^44^ respectively. Only small differences in λ (below 3%) are expected between the CeO_2_ and UO_2_ matrices, or between oxidized and reduced cerium oxide; therefore, the λ values can be considered constants within a good approximation. Taking into consideration the take-off angle,^46^ cos θ, the probing depth at 80^o^ is 0.6-0.9 nm for Ce 3d and U 4f lines. Therefore, the XPS measurements obtained at take-off angles of 0 and 80° provide information from about 14 and 2 ML, respectively. Any potential influence of the film/substrate region, e.g., electronic charge transfer or lattice strain inducing a concentration gradient of Ce^3+^ states, is not directly measured.^49^

### 3.2 Ce–U Charge Transfer and Oxygen Excess

As CeO_2_ alone shows no signs of reduction, the incorporation of U^4+^ therefore results in the formation of Ce^3+^ cations. The mechanism is accepted to involve a single charge-transfer process between a Ce and a U cation:^25–27,53^

1 $${\mathrm{Ce}}^{4+}+U^{4+}\to {\mathrm{Ce}}^{3+}+U^{5+}$$

By weighing the Ce^3+^ and U^5+^ total fractions shown in Figure 4 (*F*_Ce_^3+^ and *F*_U_^5+^, respectively) by the Ce and U content, *C* (i.e., considering the uranium fraction, *u*, relative to Ce+U), it is possible to check whether the charge transfer indeed describes the changes in oxidation states:

2 $$\Delta_{\left(0^{o},\;80^{o}\right)}=C_{{\mathrm{Ce}}^{3+}}-C_{U^{5+}}$$

2′ $$\Delta_{\left(0^{o},\;80^{o}\right)}=\left(1-u\right)F_{{\mathrm{Ce}}^{3+}}-uF_{U^{5+}}$$

Δ_(0, 80_^o^_)_^o^ is the calculated difference obtained using XPS data collected at take-off angles of 0 or 80^o^. Importantly, Δ_(0, 80_^o^_)_^o^ would be equal to 0 if for each U^4+^ oxidized to U^5+^ one Ce^3+^ is formed. As seen in Figure 5a,5b, this is not the case. The large deviation indicates that, although U cations are required, the reduction of Ce^3+^ cannot be explained solely by charge transfer. This effect is particularly pronounced at low U content, whereas the Ce^3+^ fraction decreases as the U concentration increases.

**Figure 5:**
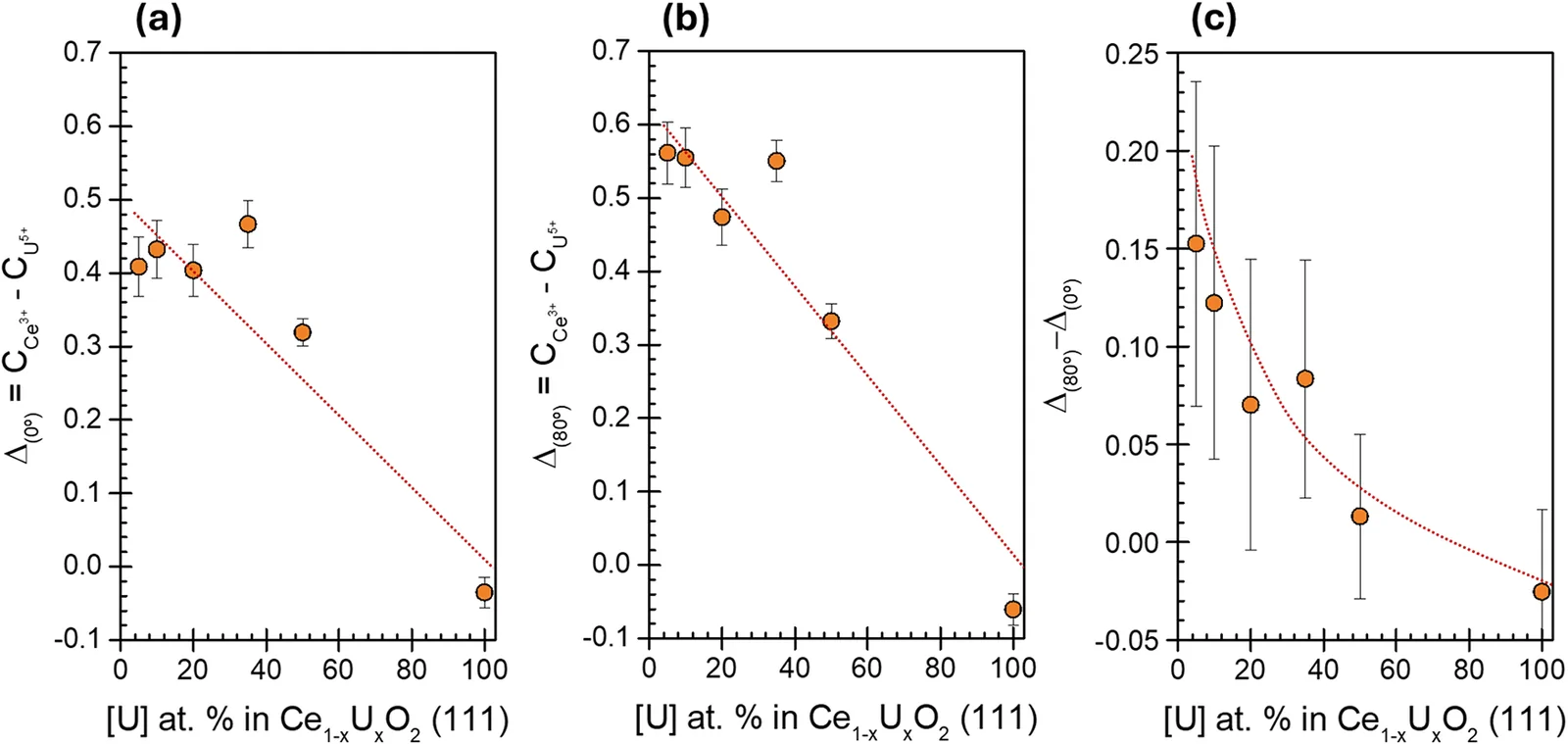
Difference, Δ, between Ce^3+^ and U^5+^ cations as extracted from XPS Ce 3d and U 4f lines as a function of the nominal amounts of U in the series Ce_1–*x*_U_*x*_O_2‑δ_ (111). (a) Δ from data collected normal to the surface, Δ _(0)_^_o_^; (b) Δ from data collected at a take-off angle of 80°, Δ_(80)_^_o_^. (c) Difference between both (a and b), Δ _(0)‑_^_o_^ Δ _(80^o^)_. The U at(%) is taken with respect to the content of Ce, excluding oxygen. The dashed red line is a guide for the eye.

#### 3.2.1 Two Different Regions for Ce^4+^ Reduction

Therefore, two regimes can be distinguished: one at low U content, where a large fraction of Ce^4+^ converts to Ce^3+^, and a second above a certain U content, where the effect becomes less pronounced. We have previously observed the same trend in a similar series, in which experimental and computational work indicated a decrease in the reduction ability of U cations at high concentrations.^26^ In terms of U content, computational results showed that the Ce local environment strongly influences the Ce–U charge-transfer process, becoming inhibited at high U concentrations (i.e., 3 U^4+^ cations in the Ce^4+^ surroundings). For lower concentrations (i.e., 2 U^4+^ cations in the Ce^4+^ environment), the computed charge transfer affected only a single Ce cation, leaving the second U cation in the 4+ state. In contrast, the presence of a single U^4+^ cation (i.e., 1 U^4+^ cation in Ce^4+^ surroundings) would require the formation of an oxygen vacancy (*V*_o_) to trigger charge transfer, resulting in two Ce^3+^ associated with V_o_ and one pair of Ce^3+^-U^5+^.

#### 3.2.2 Surface Hydroxyls Dependency on Surface Ce^3+^ States

Figure 6a displays the O 1s region, where both CeO_2_ and UO_2_ films show a prominent peak near 530.0 eV associated with lattice oxygen anions, whereas no noticeable contribution from surface hydroxyls is observed, even for measurements taken with large take-off angles. Films containing U, particularly below 35%, present a clear signal at about 1.5 eV above the main line associated with surface hydroxyls (for grazing measurements). Water in the chamber background (at about 2 × 10^–10^ mbar) dissociatively adsorbs onto Ce^3+^ cations, forming pairs of surface hydroxyls.^48^ The sticking coefficient of water on Ce^3+^–O^2–^ surface pairs is about unity,^54^ a general trend shared by most reduced metal oxides compared to fully oxidized counterparts. Therefore, the presence of hydroxyl groups is a qualitative indicator of reduced Ce^3+^, consistent with the Ce 3d signals in Figures 2 and 4.

**Figure 6:**
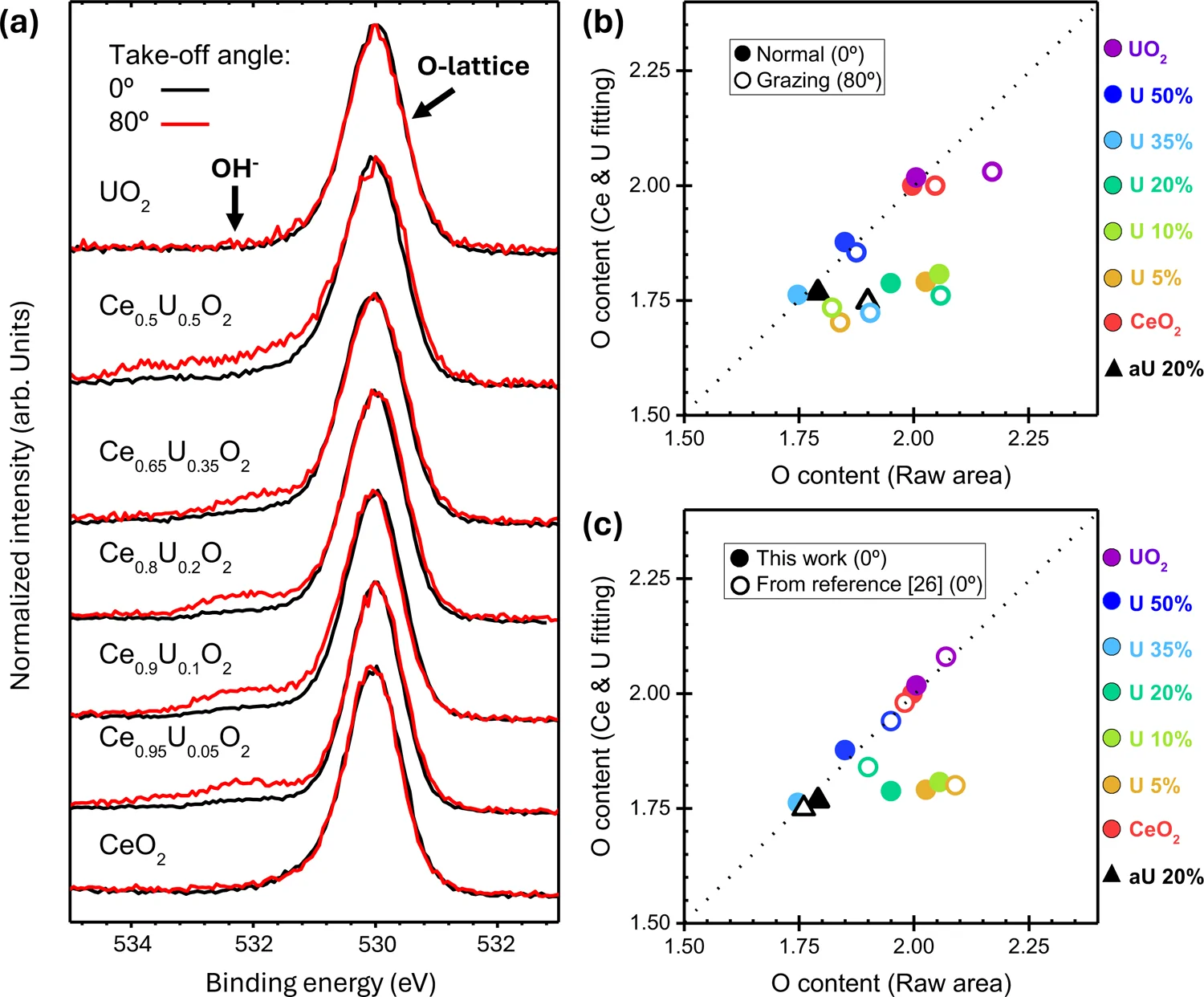
(a) XPS O 1s spectra of the series at the indicated take-off angles. (b) Computed oxygen content: *x*-axis, using atomic sensitivity factors and total intensity of O 1s, Ce 3d, and U 4f_7/2_ lines; *y*-axis, estimated from Ce^3+^/Ce^4+^ and U^4+^/U^5+^ cations (extracted from XPS fit of Ce 3d and U 4f lines), taking a (–2) oxidation state of lattice oxygen. (c) A similar plot to (b) for the series in two different runs (filled symbols from this study and open symbols are reproduced from ref 26, C. Morales et al., J. Phys. Energy, 2025, 7, 025012. Available under a CC BY 4.0 licence. Copyright 2025 The Authors. The triangle symbols in **6b** and **6c** refer to disordered, non-epitaxial thin films with 20% U content grown on amorphous Ru templates.

#### 3.2.3 Calculated Film Stoichiometry for Ce_1–*x*_U_*x*_O_2±δ_ Series

The computed oxygen-to-metal ratios in Figure 6b show almost stoichiometric CeO_2_ and UO_2_ reference films. We have computed (and compared) the oxygen-to-metal ratios of the samples in two ways. These are (i) based on the O 1s, Ce 3d, and U 4f_7/2_ total intensity corrected by the corresponding atomic sensitivity factors (*x*-axis), and (ii) based on the expected oxygen amount calculated from charge compensation (in this case, assuming O^2–^ is present solely and using the fraction of Ce^3+^/Ce^4+^ and U^4+^/U^5+^ oxidation states) (*y*-axis). It is worth noting that the presence or absence of surface hydroxyls marginally changed the numbers. Compared to the binary oxides and films with a large percentage of U (above 35%), films with lower percentages (U < 25%) show a significant deviation, with a higher O content estimated using the intensity of the O 1s line (i) than that computed assuming a -2 oxidation state for lattice oxygen anions (ii). The film grown on amorphous Ru templates, and therefore structurally disordered compared to their epitaxial counterparts, does not present any apparent deviation. These trends are not unique to this particular experiment. Figure 6c compares the current series with a previous study (ref 26), both measured in situ at a take-off angle normal to the surface. The similarity between the two runs provides further confidence in the results.

It is worth noting the differences between the surface and near-bulk regions in terms of Ce^3+^ excess and oxygen content. As shown in Figures 4 and 5, the two topmost atomic layers contain more Ce^3+^ cations and fewer U^5+^ cations than the near-bulk regions when the U content is low (< 35**%**). Figure 5c indicates a nonlinear decrease of the difference between Ce–U charge-transfer near the surface and near the bulk, tending to zero as U content increases. Considering the following equation:

3 $$\Delta \left(u\right)=\Delta_{80}-\Delta_{0}=\left(1-u\right)\left[F_{Ce3+}\left(80^{o}\right)-F_{\mathrm{Ce}3+}\left(0^{o}\right)\right]-u\left[F_{U5+}\left(80^{o}\right)-F_{U5+}\left(0^{o}\right)\right]$$

And replacing [*F*_Ce3+_(80^o^) – *F*_Ce3+_(0^o^)] by δ_Ce3+_(*u*) and [*F*_U5+_(80^o^) – *F*_U5+_(0^o^)] by δ_U5+_(*u*) to simplify, we obtain

4 $$\Delta \left(u\right)=\left(1-u\right)\delta_{\mathrm{Ce}3+}\left(u\right)-u\delta_{U5+}\left(u\right)$$

dAt low *u*, Δ­(*u*) is dominated by (1–*u*) δ_Ce3+_(*u*), whereas at higher *u* values, the second term gains weight, thus causing a curve in the falling of Δ_80_ – Δ_0_. In other words, the nonlinear decrease of the plot in Figure 5c with the overall U cations content arises because the surface cations imbalance is dominated by the Ce^3+^ contribution at low U content. The higher concentration of Ce^3+^ at the surface and the concomitant formation of surface hydroxyls can explain the higher deviations observed in Figure 6b for 80° measurements compared to those taken at 0°.

The following sections will cover the difference in the Ce^3+^ and U^5+^ fractions and oxygen content between the surface and near-bulk regions.

### 3.3 Possible Physical Interpretations

The epitaxial Ce_1–*x*_U_*x*_O_2_._δ_ (111) thin films reveal a complex picture that deviates from the commonly accepted description, which relies on direct charge transfer between the two metal cations of the Ce^4+^-U^4+^ pair. There appears to exist an interplay between a series of factors previously pointed out, but often separately in the literature: (i) the oxygen as an electronic mediator,^53^ (ii) lattice distortion due to the expansion (Ce–O) and contraction (U–O) of bond lengths,^26,53^ (iii) and an entropic contribution to the free energy that depends on cation distribution and increases with higher charge disorder.^26,27^ Taken together, these observations suggest that the Ce–U interaction is accompanied by lattice distortion and oxygen-mediated electronic redistribution involving polarons and local changes in bonding character. To rationalize these observations, the following complementary mechanisms are outlined.

#### 3.3.1 Strain-Assisted Reduction via Lattice Distortion

While the similar ionic radii of Ce^4+^ and U^4+^, 1.03 and 0.97 Å, respectively, are expected to prevent a high distortion of the fluorite lattice, multiple computational studies show an asymmetric oxygen displacement towards the uranium atoms and away from the Ce cations^27,53^ (see schematic representation in Figure 7a). Concomitantly, lattice distortion in ceria is associated with Ce–O charge transfer and the polaron-hopping mechanism, which is a phonon-induced process that involves an antiphase breathing mode where the oxygen–metal bond length expands around the Ce^3+^ cation and contracts around the oxidized site.^55,56^ Therefore, the displacement of oxygen anions and the subsequent effective charge transfer mediated by them result in Ce^3+^ and U^5+^ states that have considerably different sizes, with ionic radii of 1.14 Å (ca. 10% increase) and 0.84 Å (ca. 15% decrease), respectively. In this line, as-synthesized mixed-oxide powders exhibit lattice parameters below the linear trend predicted by Vegard’s law for CeO_2_ and UO_2_, which is attributed to the presence of U^5+^ and U^6+^ states.^25,57,58^

**Figure 7:**
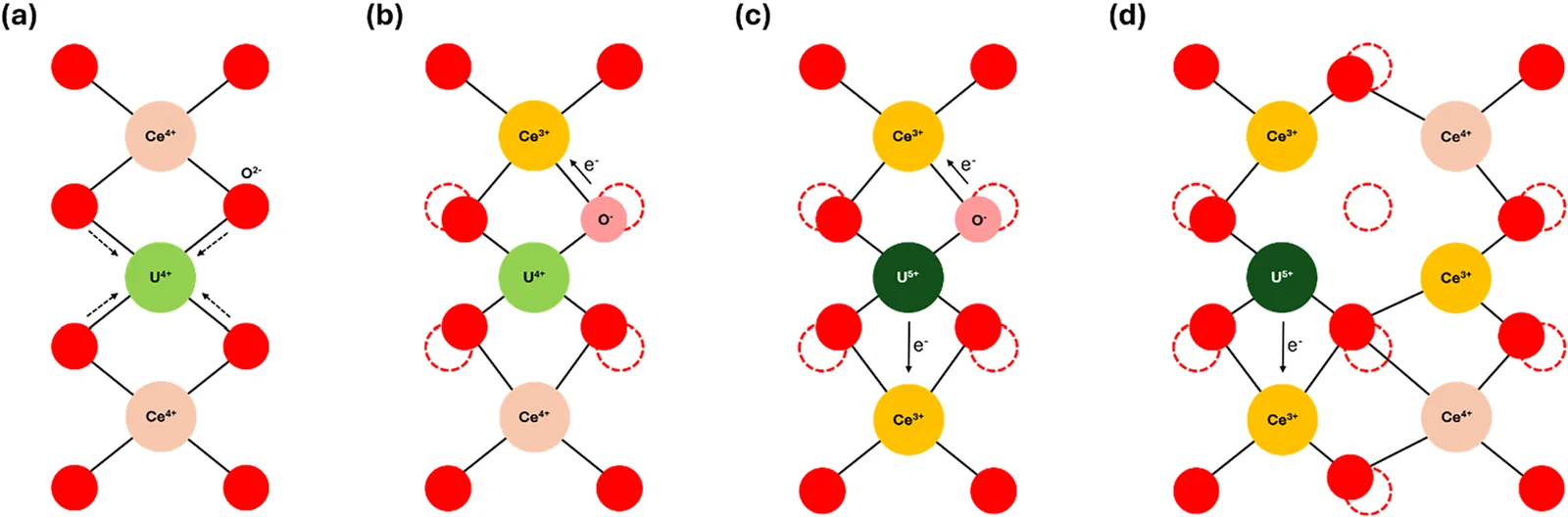
(a) Ground state of mixed Ce_1–*x*_U_*x*_O_2‑δ_ characterized by a fluorite structure where Ce^4+^ (light brown) and substitutional U^4+^ cation (light green) are surrounded by 8 O atoms each (red, here simplified to two dimensions; four O atoms). The arrows point to the displacement of oxygen lattice anions toward the U cation. Schematic representations of (b) Ce^4+^ + O_(l)_^2–^ + U^4+^ → Ce^3+^ + O_(l)_^–^ + U^4+^ (eq 6), 2 Ce^4+^ + O_(l)_^2–^ + U^4+^ → 2 Ce^3+^ + O_(l)_^–^ + U^5+^ (eq 7), and 3 Ce^4+^ + O_(l)_^2–^ + U^4+^ → 3 Ce^3+^ + V_O_ + 1/2 O_2_ + U^5+^ (eq 8). Ce^3+^ and U^5+^ states are colored in yellow and dark green, respectively. The dashed red circles represent the original position of the oxygen lattice anions, while the light red represents the O^–^ state associated with the Ce^3+^ polaron. The arrows represent the direction of the electron charge transfer.

Furthermore, this size difference of Ce^3+^ and U^5+^ cations induces additional local stress within the ceria-fluorite structure. As recently noted by us,^26^ DFT+U calculations show that the maximum strain occurs concomitantly with Ce–U charge transfer in high charge-disorder systems, with the presence of an oxygen vacancy, three Ce^3+^ cations, and one U^5+^ cation. In this case, the Ce^4+^ cations adjacent to the nearest oxygen atoms experience a large bond distance expansion, whereas the U^5+^ cation shows an asymmetric contraction of its bond distances to the seven oxygen ions (from 2.5 to 6.6%). Similar anisotropic strain of lower magnitude has been modelized for Ce^3+^ and Ce^4+^ cations around an oxygen vacancy in bare CeO_2‑x_.^59^ In short, the initial Ce–U charge transfer and associated lattice distortion via oxygen displacement can induce large strains in the fluorite structure that may promote the formation of more polarons, i.e., additional Ce^3+^ states beyond the expected Ce^4+^+U^4+^ → Ce^3+^+U^5+^. The same DFT+U calculations indicate that charge transfer can still occur, with lower lattice distortion, when two U cations are adjacent, and is inhibited when three U cations are adjacent. Therefore, the final lattice deformation depends on the local environment (not only the U content).

Importantly, ceria lattice strain is known to decrease the formation energy of oxygen vacancies, and many ceria doping strategies take advantage of this effect. For instance, the iso-valent substitution of Ce^4+^ cations by smaller ones (e.g., Zr^4+^, Pr^4+^, or Hf^4+^)^21,17,60^ induces an initial lattice compression that is relaxed by the formation of Ce^3+^ cations (and associated oxygen vacancies). Similarly, alio-valent doping with cations of lower oxidation state (e.g., La^3+^ or Sm^3+^) and different atomic radii than ceria’s critical radius (1.038 Å)^61,62^ induces the formation of Ce^3+^ states and extrinsic oxygen vacancies, i.e., due to charge compensation, while stressing the lattice, which helps in the formation of intrinsic oxygen vacancies upon thermal reduction. A recent study combining DFT and advanced surface characterization of Sm-doped ceria (111)-oriented nanoislands on Ru(0001) shows how the addition of Sm^3+^ induces ceria lattice expansion and decreases the formation energy of intrinsic oxygen vacancies,^22^ thus resulting in improved redox kinetics compared to undoped ceria.^50,63^ The oxidation step from U^4+^ to U^5+^ mirrors the same effect; the initial oxygen displacement will not only trigger the Ce–U charge transfer, but will effectively reduce the energy of oxygen vacancy formation^25^ and consequently boost the partial reduction of the mixed oxide compared to undoped CeO_2_.

#### 3.3.2 Oxygen-Mediated Charge Redistribution and Polarons

The above qualitative discussion links the charge-transfer mechanism to induced strain and, for U content above 30%, explains the excess of Ce^3+^ states (Figures 4b and 5a) above the expected value in the reduced Ce_1–*x*_U_*x*_O_2–δ_ matrix (Figure 6b). However, films with low U content (<30%) exhibit a greater Ce^3+^/U^5+^ deviation and an apparent excess of oxygen when charge compensation is achieved solely by O^2–^ lattice anions (see Figures 5a and 6b,c). For XPS measurements taken at 0°, analysis of the data after subtraction of the surface hydroxyl contribution largely yielded results similar to those obtained without subtraction, suggesting that OH^‑^ groups are not the origin of this deviation in mixed films. Moreover, the possible formation of superoxo (O_2_^−)^ and peroxo (O_2_^2–^) species, resulting from the reaction of molecular oxygen via electron transfer with a 2·Ce^3+^-V_o_ complex,^64–69^ is unlikely due to the very low O_2_ partial pressure at a base pressure of 3 × 10^–10^ mbar (as indicated by others from ambient pressure XPS measurements^70^). Besides, the formation of superoxo or peroxo species will yield Ce^4+^ cations that can only be associated with two lattice O^2–^, which is accounted for on the *Y*-axis of Figure 6b,c plots.

One plausible explanation is that not all oxygen anions have a -2 oxidation state; some lattice oxygens are in a -1 oxidation state, or even neutral. This would be driven by the above-discussed oxygen displacement towards the U cations and the concomitant lattice deformation, likely resulting in a change in bonding nature from ionic to a more covalent type, spreading the electron density around the atoms. Therefore, the initial U–O bond length contraction and Ce–O expansion could result in charge transfer between oxygen and cerium, i.e., polaron coupling between the Ce^3+^ and O^–^ states, while keeping a U^4+^ center unchanged (Figure 7b):

5 $${\mathrm{Ce}}^{4+}+{O^{2-}}_{\left(l\right)}+U^{4+}\to {\mathrm{Ce}}^{3+}+{O^{-}}_{\left(l\right)}+U^{4+}$$

An equivalent one-electron transfer from the lattice oxygen O 2p states to the Ce 4f orbitals (hence an electron trapped near a Ce^4+^/Ce^3+^ that effectively reduces the ceria cation) has been proposed in the interpretation of electron paramagnetic resonance (EPR) spectra on reduced CeO_2_,^38^ while electron irradiation experiments showed that displacement damage processes provoked the induction of Ce^3+^ states in ceria single crystals without structural modifications in the fluorite structure.^71^ Similarly, small perturbations in a non-defective ceria matrix, such as UV light absorption,^72,73^ can also induce such a exciton-polaron complex, consisting of an electron polaron on a cerium atom (Ce^3+^) adjacent to one hole polaron on one or more oxygen atoms (O^–^). The dynamics of polarons are, in fact, responsible for the electronic transport and oxygen migration in ceria^56^ and are correlated with its redox properties and catalytic behavior,^74,75^ as well as those of related compounds.^76^ Moreover, an effective increase in the oxidation state of oxygen anions (from -2 to -1) around the uranium center could help minimize their mutual secondary electronic repulsion, because a consequence of the U–O bond length contraction is a decrease in the O–O distance. The O 1s binding energy of such a species is expected to be very close to that obtained upon H_2_O_2_ dissociative adsorption (–O–OH),^77^ which is about 532.5 eV. Given the weak signal in this region, Figure 6a, when compared to that of the lattice O 1s and the limit of the resolution of the spectrometer, it is not possible to assess its presence.

This charge-transfer mechanism, mediated by the U^4+^ cation, is not the only candidate. A displaced oxygen lattice could mediate the electron transfer as per:

6 $$2{\mathrm{Ce}}^{4+}+{O^{2-}}_{\left(l\right)}+U^{4+}\to 2{\mathrm{Ce}}^{3+}+{O^{-}}_{\left(l\right)}+U^{5+}$$

In this case, the second Ce^3+^ would be associated with a Ce–U charge transfer, thus stabilizing a U^5+^ state (Figure 7c). As previously noted, a final variation of this scenario would be the production of the oxygen vacancy and the formation of three Ce^3+^ states and one U^5+^ (Figure 7d), resulting in an asymmetric lattice deformation as shown by DFT+U calculations:^26^

7 $$3{\mathrm{Ce}}^{4+}+{O^{2-}}_{\left(l\right)}+U^{4+}\to 3{\mathrm{Ce}}^{3+}+V_{O}+1/2O_{2}+U_{5+}$$

Despite the absence of theoretical simulations that directly support these qualitative explanations of the experimental results, the trends observed for diluted systems, i.e., U content below 30%, can be explained by a combination of the three scenarios summarized by eqs 6–8.

Considering the role of the entropic factor in terms of structural and charge disorder, diluted systems would favor conditions of higher charge transfer and lattice deformation than at higher U concentrations, where the probability of finding two or more substitutional cations as close neighbors is higher.^26,27^ The strong background and faint diffraction spots in LEED is consistent with high stress levels and defect concentration in U-diluted Ce_1–*x*_U_*x*_O_2–δ_ (111) thin films, while the strong Ce^3+^/U^5+^ deviation and excess of oxygen support the role of Ce^3+^.···O^–^ polaron clusters and therefore that of oxygen as a mediator in the Ce/U charge-transfer process.^53^

Notably, epitaxial and amorphous-like Ce_0.8_U_0.2_O_2‑δ_ films exhibit significant differences in oxygen content: amorphous-like Ce_0.8_U_0.2_O_2‑x_ films appear to only present O^2–^ anions (see Figure 6b,c). In this work it is found that the local environment of an ordered fluorite structure promotes scenarios described by eqs 6 and 7 mediated by charge transfer, whereas a disordered ceria environment promotes reduced films via oxygen vacancy formation (eq 8). It is worth mentioning that an oxygen vacancy is one possible source of the electrons that reduce Ce^4+^ to Ce^3+^, but it is not a prerequisite for the formation of a Ce-centered electron polaron. In the Ce–U system, Ce^3+^ species can also be generated by cation–cation charge transfer, U^4+^ + Ce^4+^ → U^5+^ + Ce^3+^, without oxygen removal. These differences in how Ce–U charge transfer manifests, depending on the local structural and chemical environment, together with differences in synthesis routes, morphology (i.e., thickness of thin films or size of nanoparticles), and post-treatments, explain the complexity of a unified picture of the reduced state properties of CeO_2_ as reported in numerous work.^26,53,78–83,37^

Given the key role of the surface in catalytic and related processes, further comparative studies of the surface and bulk using simultaneous local chemical and structural experimental techniques are required to complete the picture outlined in this section.

## 4 Conclusions

Epitaxial Ce_1–_ *_x_* U*_x_* O_2_(111) thin films exhibit reduction behavior that cannot be explained solely by a direct charge-transfer mechanism between neighboring Ce^4+^ and U^4+^ cations. Angle-resolved XPS measurements demonstrate that the concentration of Ce^3+^ states systematically exceeds that of U^5+^ states, particularly at low uranium concentrations and in the near-surface region. The deviation decreases with increasing U content, indicating a strong dependence on the local cationic environment. Quantitative oxygen-balance analysis further reveals that the measured oxygen content is inconsistent with a model in which all lattice oxygen remains in the O^2–^ state. Together with the absence of this behavior in structurally disordered films, these observations support the participation of oxygen-mediated electronic states associated with lattice distortion and polaron formation. In this framework, oxygen anions act as electronic mediators in the Ce–U interaction. The results indicate that local strain induced by the coexistence of expanded Ce^3+^ and contracted U^5+^ coordination environments promotes additional reduction pathways beyond direct Ce–U charge transfer.

## Supplementary Material



## Notes

Factor analysis (FA) of Ce 3d and U 4f regions, inelastic peak shape analysis (IPSA) of the XPS O 1s, and estimated film composition from XPS Ce 3d, O 1s, and U 4f lines (PDF)
