# Supplementary material for: Beyond Ce–U Charge Transfer: Strain and Polarons in Epitaxial Ce1– x U x O2(111)
Source: J Phys Chem C Nanomater Interfaces. 2026 Sep 17;130(39):13556–67. doi: 10.1021/acs.jpcc.6c05108 (PMC13637078; doi:10.1021/acs.jpcc.6c05108)
Supplement: Supplementary file 1 [file jp6c05108_si_001.pdf]

Supporting Information

Beyond Ce–U Charge Transfer: Strain and Polarons in Epitaxial $Ce_{1-x}U_xO_2(111)$

Carlos Morales[1], Rudi Tschammer[1], Aman Baunthiyal[2], Thomas Gouder[3], Jan Ingo Flege[1*], and Hicham Idriss[4*]

[1]Applied Physics and Semiconductor Spectroscopy, Brandenburg University of Technology Cottbus–Senftenberg, Konrad-Zuse-Strasse 1, D-03046 Cottbus, Germany.

[2]Institute of Solid State Physics, University of Bremen, D-28359 Bremen, Germany

[3]European Commission, Joint Research Centre (JRC), Postfach 2340, D-76125 Karlsruhe, Germany.

[4]Department of Chemistry, University College London, UCL, London, WC1E 6BT, UK.

*Corresponding authors:

Jan Ingo Flege: JanIngo.Flege@b-tu.de

Hicham Idriss: h.idriss@ucl.ac.uk

Factor Analysis (FA) of Ce 3d and U 4f regions

Main components

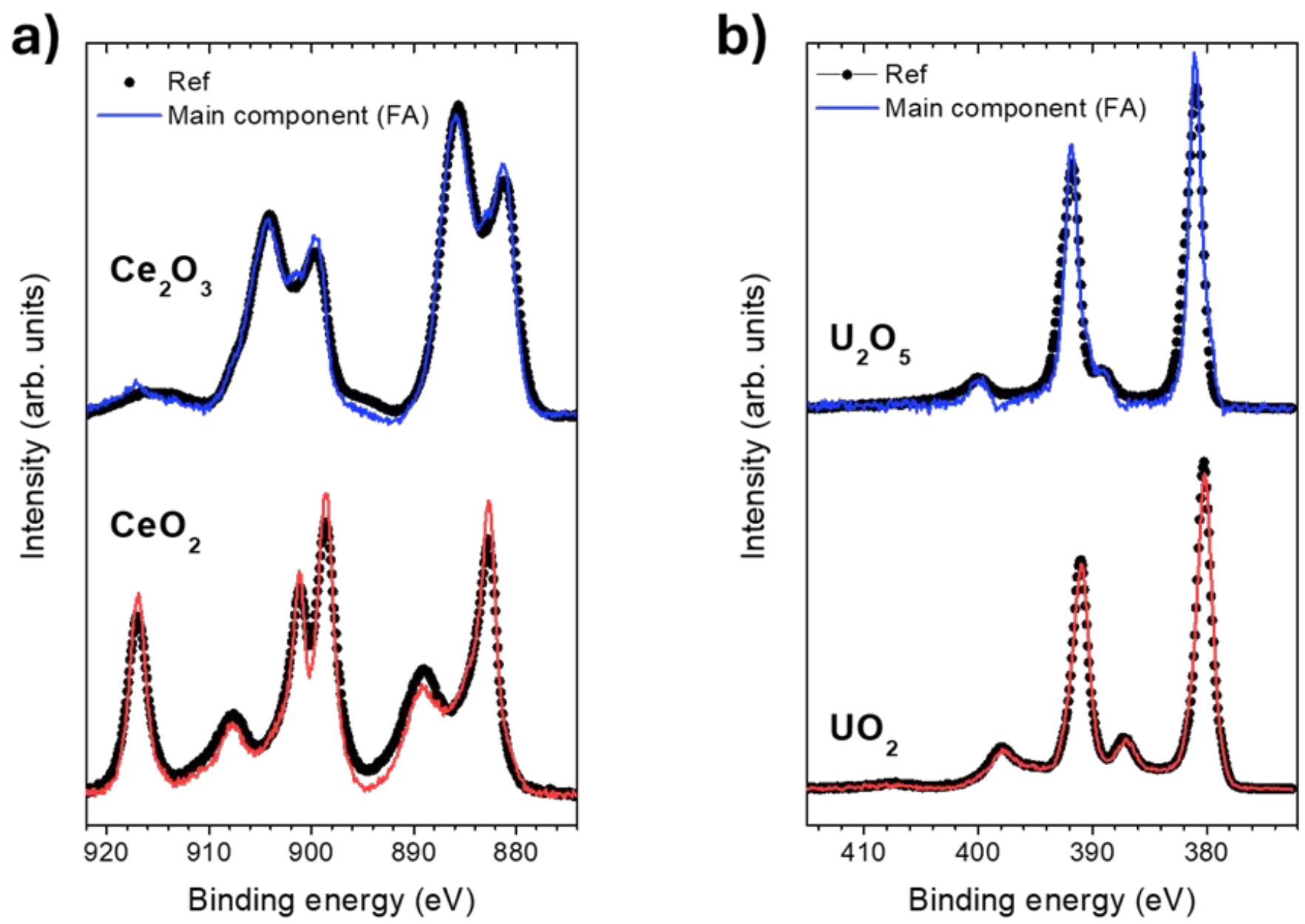

Figure S1. Comparison between reference XPS spectra (black full symbols) and main components obtained from target testing factor (continuous blue and red lines) analysis for **(a)** Ce 3d and **(b)** U 4f regions.

Main components

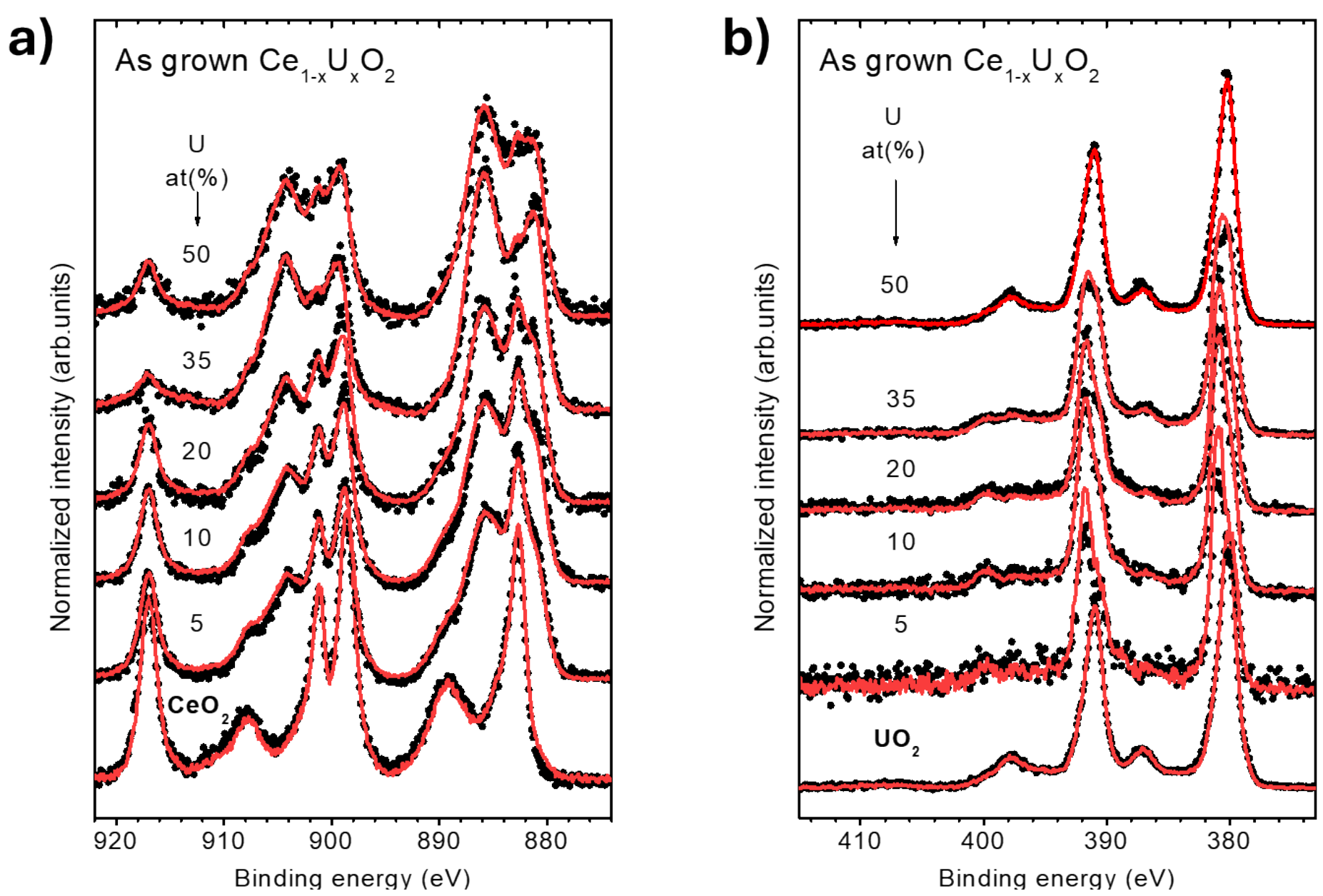

Figure S2. Example of FA fitting (red) for the **(a)** XPS Ce 3d and **(b)** XPS U 4f of the as-grown samples (raw data, black circles), take-off angle 0° for the series $Ce_{1-x}U_xO_2$.

Inelastic peak shape analysis (IPSA)

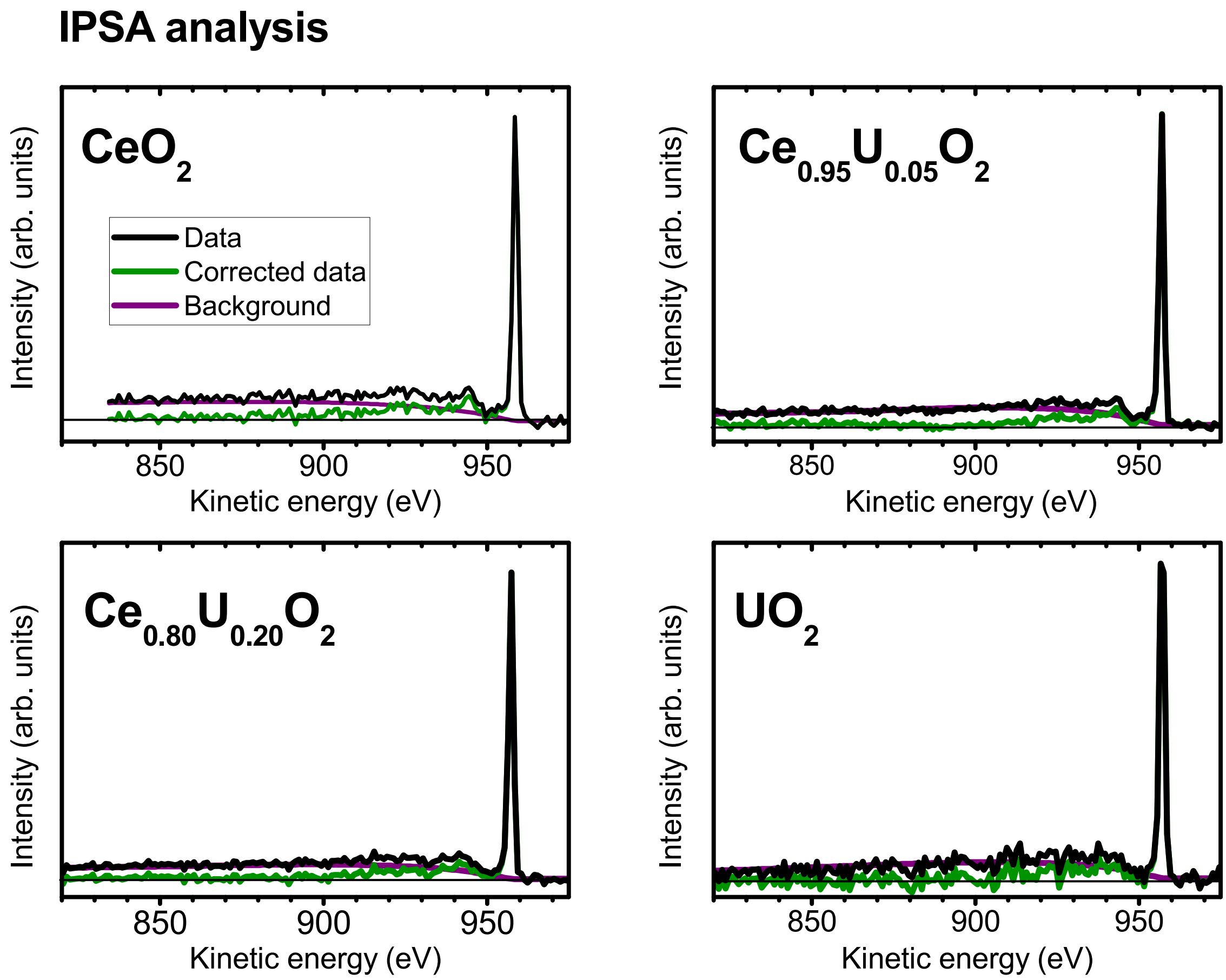

Figure S3. Inelastic peak shape analysis (IPSA) performed with QUASES-Analyze software using a buried layer model (i.e., continuous film without top overlayer) on the O 1s region for four exemplary analyses (CeO_2, $Ce_{0.95}U_{0.05}O_{2\pm\delta}$, $Ce_{0.8}U_{0.2}O_{2\pm\delta}$, and UO_2) of (111)-oriented films grown on $Ru(0001)/Al_2O_3(0001)$ substrates.

Sample	d (Å)	Sample	d (Å)
$Ce_{0.8}U_{0.2}O_2$	80 ± 5	a-$Ce_{0.8}U_{0.2}O_2$	80 ± 5
$Ce_{0.9}U_{0.1}O_2$	80 ± 5	UO_2	80 ± 5
$Ce_{0.95}U_{0.5}O_2$	80 ± 5	$Ce_{0.5}U_{0.5}O_2$	80 ± 5
CeO_2	85 ± 5	$Ce_{0.65}U_{0.35}O_2$	70 ± 5

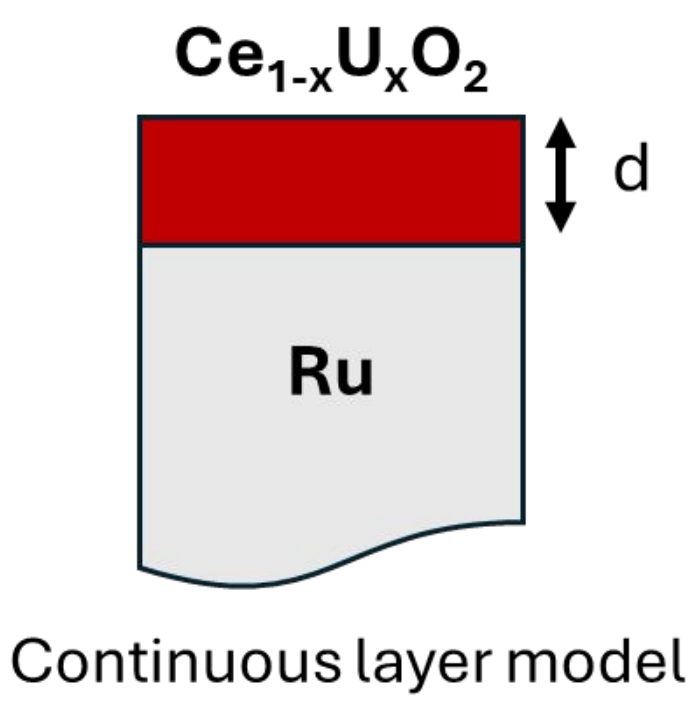

Table S1. Estimated thickness from IPSA analysis. For comparison purposes, an amorphous (nanocrystalline, a-$Ce_{0.8}U_{0.2}O_{2\pm\delta}$) grown under the same conditions on amorphous Ru prepared on SiO_2 substrates is shown. The left panel shows an scheme of the used model in the IPSA analysis.

Sample composition estimated from XPS

Table S2. Estimated composition of as-grown samples by XPS measurements at two take-off angles (0° and 80°). Atomic sensitivity factors for Ce 3d, U $4f_{7/2}$, and O 1s are 10.40, 9.20, and 0.75, respectively. An overall uncertainty of ±2% is estimated for the at% calculation.

		**Intensity**			**Atomic concentration**			**$Ce_{1-x}U_xO_y$**		
**Sample**	Take-off angle (°)	Ce 3d (cps)	U 4f (cps)	O 1s (cps)	Ce at(%)	U at(%)	O at(%)	1-x	x	y
CeO_2	0	139637.5	0	20131.43	33.4	0	66.6	1	0	2.00
	80	46400.59	0	6858.072	32.8	0	67.2	1	0	2.05
$Ce_{0.95}U_{0.05}O_{2\pm\delta}$	0	214037.8	14198.68	32662.17	31.6	1.4	67.0	0.96	0.04	2.03
	80	58632.78	1947.677	7956.89	34.5	0.7	64.8	0.98	0.02	1.84
$Ce_{0.9}U_{0.1}O_{2\pm\delta}$	0	190572.9	48084.33	32902.76	28.1	4.6	67.3	0.86	0.14	2.06
	80	61209.39	12783.25	9140.237	31.2	4.2	64.6	0.88	0.12	1.82
$Ce_{0.8}U_{0.2}O_{2\pm\delta}$	0	144793.5	51414.61	23021.02	29.1	6.7	64.2	0.81	0.19	1.79
	80	62564.89	24640.27	10766.19	27.5	7.0	65.5	0.80	0.20	1.90
$Ce_{0.65}U_{0.35}O_{2\pm\delta}$	0	176328	142887.7	33899.21	23.9	12.5	63.6	0.66	0.34	1.75
	80	47036.12	38718.22	9912.614	22.5	11.9	65.6	0.65	0.35	1.90
$Ce_{0.5}U_{0.5}O_{2\pm\delta}$	0	97252.84	205894.3	30757.85	14.8	20.3	64.9	0.42	0.58	1.85
	80	26382.97	47018.95	7685.049	16.2	18.6	65.2	0.46	0.54	1.88
UO_2	0	0	273644.9	25597.32	0	33.3	66.7	0	1	2.01
	80	0	88105.59	8921.798	0	31.5	68.5	0	1	2.17
amorphous $Ce_{0.8}U_{0.2}O_{2\pm\delta}$	0	144793.5	51414.61	23021.02	29.1	6.7	64.2	0.81	0.19	1.79
	80	62564.89	24640.27	10766.19	27.5	7.0	65.5	0.80	0.20	1.90
